# Type-2 epithelial-mesenchymal transition in oral mucosal nonneoplastic diseases

**DOI:** 10.3389/fimmu.2022.1020768

**Published:** 2022-10-31

**Authors:** Zhaosong Meng, Tianle Yang, Dayong Liu

**Affiliations:** ^1^ Department of Oral and Maxillofacial Surgery, Tianjin Medical University Stomatology Hospital, Tianjin, Tianjin, China; ^2^ School of Stomatology, Tianjin Medical University, Tianjin, China; ^3^ Department of Endodontics & Laboratory for Dental Stem Cells and Endocrine Immunology, Tianjin Medical University School of Stomatology, Tianjin, China

**Keywords:** epithelial-mesenchymal transition, craniofacial embryogenesis, oral mucosa alterations, keloid, fibrosis, immunological microenvironment

## Abstract

The oral mucosa is a membranous structure comprising epithelial and connective tissue that covers the oral cavity. The oral mucosa is the first immune barrier to protect the body against pathogens for systemic protection. It is frequently exposed to mechanical abrasion, chemical erosion, and pathogenic invasion, resulting in oral mucosal lesions, particularly inflammatory diseases. Epithelial-mesenchymal transition (EMT) is a crucial biological process in the pathogenesis of oral mucosal disorders, which are classified into three types (types 1, 2, and 3) based on their physiological consequences. Among these, type-2 EMT is crucial in wound repair, organ fibrosis, and tissue regeneration. It causes infectious and dis-infectious immunological diseases, such as oral lichen planus (OLP), oral leukoplakia, oral submucosal fibrosis, and other precancerous lesions. However, the mechanism and cognition between type-2 EMT and oral mucosal inflammatory disorders remain unknown. This review first provides a comprehensive evaluation of type-2 EMT in chronically inflammatory oral mucosal disorders. The aim is to lay a foundation for future research and suggest potential treatments.

## Introduction

**Figure 1:**
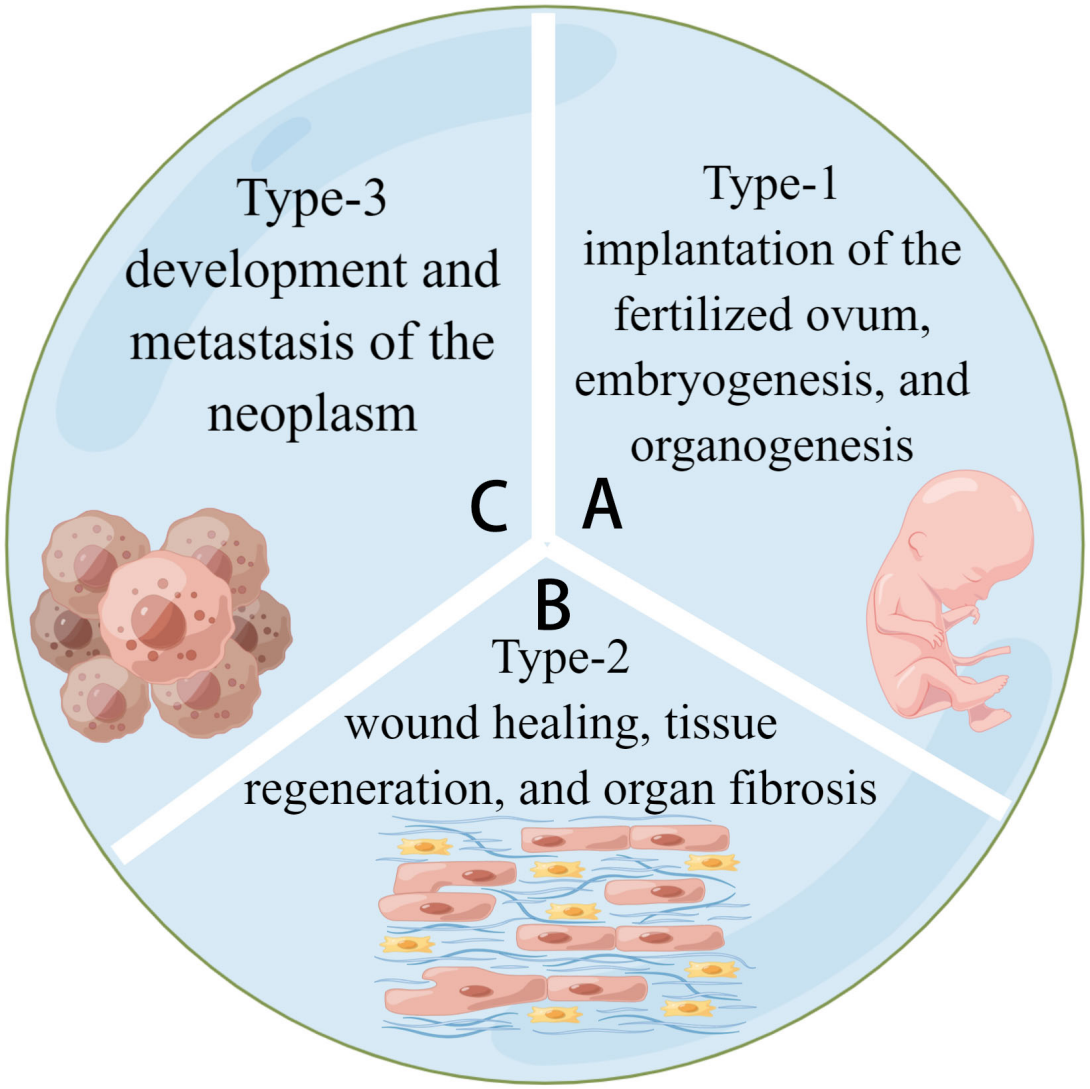
Three types of EMT. **(A)** Type-1 EMT is related to the embryogenesis of tissues and organs. **(B)** Type-2 EMT is related to tissue regeneration and fibrosis. **(C)** Type-3 EMT is related to tumor development and metastasis. They share a common model of cell activities.

In the 1980s, epithelial-mesenchymal transition (EMT) was identified as a feature of embryogenesis (1). Under different stimuli, epithelial cells lose polarity and cell-cell junctions and thus gain the ability to migrate, transforming into spindle-like mesenchymal cells. The reverse process of EMT is known as a mesenchymal-epithelial transition (MET). Both are crucial biological processes in embryonic development and tissue genesis in the dynamic balance of alteration (2). (**Figure 1**) Whether EMT or MET describes a process, quasi-mesenchymal cells are a type of transitional cell with characteristics of both epithelial and mesenchymal cells. Cancer metastasis and invasion have been linked to hybrid cells. They undergo partial EMT and have unique properties such as collective cell migration (3).

**Figure 2:**
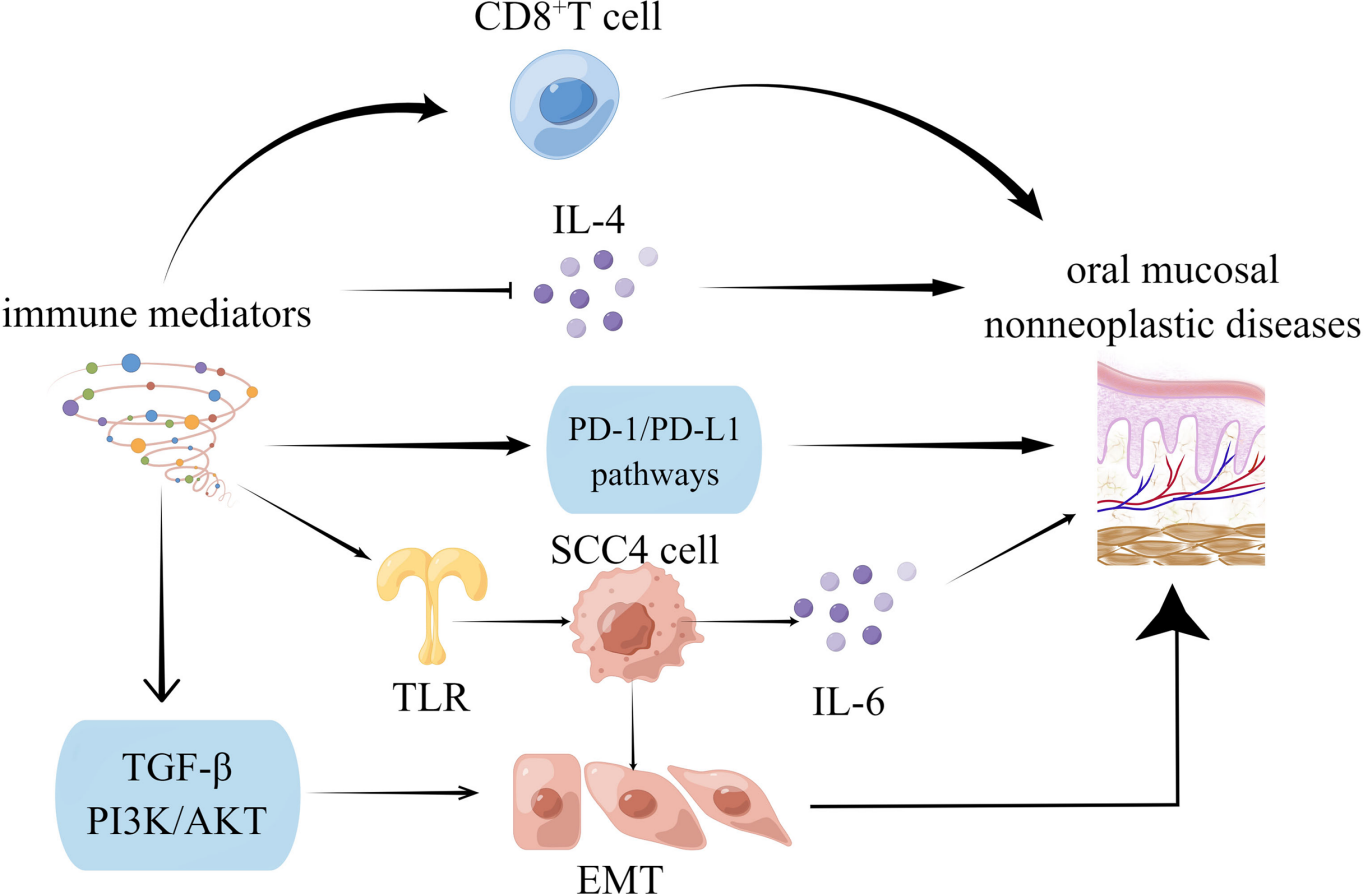
Oral mucosal non-neoplastic diseases were mediated by immune microenvironment.

EMT is classified into three types based on the biological environment in which it occurs: types 1, 2, and 3. Types 1 and 2 EMTs are associated with embryonic, regenerative, and pathological processes, respectively, with no abnormal cell proliferation (4, 5). Type-3 EMT is critical for tumor development and metastasis because it allows cancerous cells to generate, develop, and spread (6). (See **Figure 2**) Most current research focuses on Type-3 EMT, which is responsible for tumor genesis. However, types 1 and 2 EMTs have received little attention. Types 1 and 2 EMTs conclude the biological development of craniofacial tissues and organs from birth and the pathological processes of abnormal transition after the individual fully develops.

Fibrosis of the oral mucosa occurs in the inflammatory microenvironment of type-2 EMT. The immune microenvironment regulates cytokines and molecules in some signaling pathways, and the mechanisms are systematically generalized. Scientists have worked on EMT to treat degenerative diseases, repair injuries, rebuild tissue and organs, and delay senescence (7).

This review summarized the function and mechanism of type-1 and type-2 EMTs in oral mucosal nonneoplastic diseases. We proposed potential therapies for EMT-related diseases to lay a foundation for future clinical use in stomatology.

## Type-2 EMT in oral mucosa

The oral cavity is protected by oral mucosa, primarily squamous epithelium, an immune system component. This review highlighted the oral mucosa as the human body’s immune barrier to defend against pathogens for systematic protection. Innate and adaptive immunity combine to form oral mucosal immunity. The former includes the physical barrier of mucosal epithelium, which excretes defensin, interleukin (IL)-8, and tumor necrosis factor-α (TNF-α); the normal flora, which alters the surrounding environment to inhibit the growth of potential pathogens; and the innate immune cells such as dendritic, Langerhans, and mast cells. The latter is known as the Mucosal Immune System (MIS). It produces secretory IgA in the Waldeyer’s ring and connects the inducer and effector sites *via* cell homing. MIS is involved in the local adaptive immune response and can cause mucosal cytotoxicity.

Type-2 EMT occurs in wound healing, tissue regeneration, and organ fibrosis and may result in keloids in the repair of the human epithelium in the inflammatory microenvironment. In the oral mucosa, it has been reported that simple wound healing causes no scar, which differs from the skin (8). However, certain microbiomes and tobacco and alcohol use can cause the pathological formation of fibrosis and keloids in the oral mucosa *via* EMT, which may delay the ultimate healing of the oral mucosa. There is ample space for further investigation in this area, the details of which remain unknown.

### EMT in oral mucosal wound repair and regeneration

When a wound occurs, the skin and mucosa go through hemostasis, inflammation, proliferation, and remodeling. Granulation tissue forms in the inflammatory microenvironment and then progresses to the proliferation stage, where keratinocytes and fibroblasts migrate to the wound bed. The former is in charge of barrier reconstruction, while the latter is in charge of secreting extracellular matrix and remodeling granulation tissue (9).

Oral health has long been closely linked to systemic health but has received little attention. The oral cavity, located at the beginning of the human digestive tract, is critical for mastication, digestion, pronunciation, and aesthetics. Even though the oral mucosa is often exposed to mechanical abrasion and tension, it heals much faster with less scarring than the skin (10). The reason might be that oral mucosal fibroblasts and dermal fibroblasts have different cell behaviors and responses to growth factors. When exposed to transforming growth factor-β (TGF-β1), oral mucosal fibroblasts have a higher average proliferation rate, a lower shrinkage capacity, and synthesize more collagen (11).

Type-2 EMT is an after-birth reactivation recognized as a way to control inflammation and tissue regeneration. In recent decades, scientists have committed to identifying the factors initiating EMT. One explanation for the phenomenon is that in acute and mild trauma, wounded epithelial cells differentiate into fibroblast-like cells to reproduce tissues and organs, which is a reparative biological process (6). However, in the case of long-term continuing inflammation, the keloid is considered pathological fibrosis. It ceases once the repair is completed (12). The interaction between TGF-β1 and pro-inflammatory cytokines can generate a microenvironment for autoregulatory loops to strengthen the EMT. Polyriboinosinic: polyribocytidylic acid (Poly (I: C)) has been shown to accelerate collective HaCaT cell migration *via* autocrine/paracrine IL-8 secretions and EMT (13). It promotes leukocyte accumulation and improves chemokine expression during wound healing (14). Poly I: C induced IL-8 production by keratinocytes by stimulating Toll-like receptor 3 (TLR3), and TLR3 is a component of wound healing in regulating inflammation, during which NF-κB is activated (15). Poly I: C can stimulate EMT and improve wound healing (16). Interestingly, it has also been found that excessive poly I: C stimulation contributes to delayed wound healing (17). The complicated mechanisms of the double effect of Poly I: C remain to be investigated further. Another explanation for activating EMT during a wound in the mucosa is that the loss of apical-basal polarity can initiate the transition. It has been demonstrated that normal apical-basal cell polarity inhibits EMT *via* SNAI1 degradation mediated by the PAR complex (18). This points to the potential role of the cell-cell junction in regulating EMT.

### EMT with oral mucosal dis-infectious diseases

Pathological processes associated with type 2 EMT include abnormal metastasis, keloid formation, and fibrosis. Scientists have reported the formation of keloids related to TGF-β1, EGF, and fibroblast growth factor (FGF) signaling pathways (19). Keratinocytes and fibroblasts influence the keloid and fibrosis of abnormal tissue, causing oral leukoplakia (OLK) or oral submucous fibrosis (20). Fibrosis has also been linked to inflammation.

Oral leukoplakia (OLK) is the most common underlying precancerous lesion and potentially malignant disorder (21). EMT can cause OLK to progress into oral squamous cell carcinoma (OSCC), linked to smoking (22) and chewing tobacco (23). It has been observed that in non-smokers, OLK occurs in conjunction with an immunosuppressive microenvironment established by activation of the PD-1/PD-L1 pathway and recruitment of CD163+ tumor-associated macrophages (TAMs), which may function in the early and transforming stages of oral tumorigenesis. The findings demonstrate that EGFR and WNT pathway proteins are overexpressed in all OLK samples, triggered by chewing tobacco, and may be a risk factor for the type of proliferation. Remarkably, the lncRNA oral leukoplakia progressed associated 1(LOLA1) has been found to promote oral mucosa epithelial migration, invasion, and EMT *via* the AKT/GSK‐3β pathway, thereby accelerating the progression of OLK (24). Elevated levels of some novel biomarkers, such as Snail and Axin2, with a high correlation to OLK malignant transformation, can predict oral tumorigenesis (25).

Oral submucous fibrosis (OSF) develops in a constant pro-inflammatory environment and has the characteristics of tissue fibrosis and degeneration diseases in various tissues and organs (26). Patients with OSF have difficulty opening their mouths and have stiff oral mucosa. It is caused by EMT, which causes oral submucous fibrosis (27). Arecoline is known as the pathogenic factor of OSF and has been shown to increase Twist expression (28).Chewing areca causes microtrauma and activates a protective inflammatory response, releasing many growth factors such as TGF-β, platelet-derived growth factor, basic FGF, and cytokines such as IL-6 and TNF-α, which promote fibrosis (29). Hinokitiol has been shown to downregulate Snail, lowering a-SMA expression and myofibroblast properties as an anti-fibrosis agent (30).

The World Health Organization classifies OLP as a premalignant chronic inflammatory disease mediated by T-lymphocytes. Smad 3 expression in OLP is higher and statistically significant than in normal oral mucosa, consistent with apoptosis, inflammation, and EMT functions (31, 32). It has also been reported that in OLP, claudin‐1, claudin‐4, and E‐cadherin are downregulated, disrupting the epithelial barrier and causing T‐lymphocytes to migrate into epithelial cells (33). Furthermore, OLP liquefaction degeneration is an EMT result primarily induced by IFN-γ, which can improve the malignant transition (34). The current studies also illustrated that the submucosal infiltration of T and B lymphocytes is more distinct in OLP than in OLK, and the immunological response is also stronger in OLP (35).

### EMT with oral mucosal infectious diseases

The oral cavity is a huge reservoir for microorganisms to grow, develop and manipulate. Millions of viruses, bacteria, and fungi colonize the mucosa epithelium forming a balanced biofilm. Once the equilibrium is disrupted, opportunistic pathogens take over and cause continuous inflammatory reactions. Oral microbiota has been found to manipulate cell migration by modulating the EMT process in such an inflammatory microenvironment. Microbiota degrades epithelial tight junction proteins, improves mesenchymal properties, and induces partial or complete EMT (36). It is frequently associated with oral mucosa infectious diseases such as gingivitis, oral candidiasis, herpes, and others. Pathogens in the oral cavity cause disease *via* different regulatory mechanisms of the EMT.

*Porphyromonas gingivalis* (*P. gingivalis*) degrades E-cadherin to regulate the epithelial function of the barrier (37). *NNMT*, *CCAT1*, and *GAS6* genes are involved in cell migration and invasion. These gene’s messenger RNA (mRNA) levels are high in *P. gingivalis*-infected oral epithelial cells (38). It also modulates the β-catenin pathway and uncouples the β-catenin destruction complex in gingival epithelial cells, facilitating nuclear translocation to activate TCF/LEF promoter elements in the following step (39).

*Streptococcus gordonii* suppresses FOXO1 and activates the TAK1-NLK negative regulatory pathway for ZEB2 induction resistance (40). Upregulation of partial EMT genes has been observed in *Fusobacterium nucleatum*-infected OSCC cells (41). The signal transducer and activator of the transcription-3 signaling pathway is activated, increasing the expression of EMT-associated genes such as *E-cadherin*, *Snail*, and *Twist*. The EMT has been widely debated over the years, particularly its role in cancer progression. However, the significance of EMT in embryogenesis, tissue regeneration, and fibrosis is rarely discussed. This review discusses the types 1 and 2 EMTs in craniofacial tissues and organs. Related disorders such as palatal cleft, dental defect, OLK, and OSF are also evaluated. There remains a long way to go to reduce the negative effects of EMT, such as the formation of keloid and fibrosis and the facilitation of neoplasm to provide theoretical support for the following research and applications of types 1 and 2 EMTs so that experimental trials of EMT can be used in the clinic and theoretical knowledge can transform from bench to bedside. Certain bacteria, lower PH, signaling molecules, loss of apical-basal polarity, and other approaches have been used to activate EMT. The EMT process, particularly type-2 EMT, strongly correlates with inflammation regulated by the immunological microenvironment. However, there remains a long way to go before determining the complete blueprint of the crosstalk among various cytokines and signaling pathways. As previously stated, we have concluded complicated mechanisms of types 1 and 2 EMTs. Many details of regulation and alteration remain unknown. It may be important for researchers to investigate the differential expression of cytokines and signaling pathways during both biological and pathological processes of EMT activities (**Table 1**).

**Table 1:** The mechanisms of Type-2 EMT in biological and pathological processes.

Type-2 EMT		Relative Cytokines	Signaling Pathways
Wound repair and tissue regeneration		TGF-β1 (11), pro-inflammatory cytokines (13),IL-8 (13),SNAI1 (18)	NF-κB (15), TGF-β (11)
Dis-infectious diseases	OLK	CD163+ TAMs (22, 23), lncRNA LOLA1 (24),Snail (25),Axin2 (25)	TGF-β1, EGF (19), FGF (19), PD-1/PD-L1 (22, 23), WNT (42), AKT/GSK‐3β (24)
	OSF	Twist (28),TGF-β (29), TNF-α (29),PDGF (29),bFGF (29), IL-6 (29),Snail (30),a-SMA (30)	
	OLP	IFN-γ (34),Smad3 (31, 32),claudin‐1 (33), claudin‐4 (33),E‐cadherin (33)	
Infectious diseases	P. gingivalis	NNMT (38),CCAT1 and GAS6 (38), TCF/LEF promoter (39),E‐cadherin (41),Snail (41), Twist (41)	β-catenin (39)
	S. gordonii		FOXO1 (40), TAK1-NLK negative regulatory pathway (40)
	F. nucleatum		STAT3 (41)

### EMT with immune regulation of the oral mucosa

Oral mucosal disease, particularly oral mucosal precancerous lesions, has been linked to changes in the immune microenvironment. The infiltration of high-grade CD8+ lymphocytes within the epithelium was linked to increased in remission rates (43). Intraepithelial CD8+ lymphocytes are likely to serve as a biomarker of remission and a potential area of biomedical research regarding OLP’s etiology and premalignant potential. The host immune system may bypass PD-L1-expressing dysplastic epithelial and recruited subepithelial cells in oral precancerous lesions. Furthermore, by inhibiting the PD-1/PD-L1 pathways, oral precancerous lesions can be prevented from transforming into cancer, and advanced cancer can be treated (44). According to previous research, OLP lesions are caused by IL-4, which is produced by several factors. It also affects various cells, resulting in OLP lesions (45).

Immune mediators are not only directly linked to precancerous lesions, but they are also indirectly mediated by EMT. It has been reported that activated oncogenic Ras post-transcriptionally enhances premalignant cell mutations, intensifying malignancy and cell invasion. There is a significant change in mRNA levels, which correlates with protein abundance and is consistent with EMT. These proteins also changed following Ras transformation, suggesting that premalignant cells were primed to become malignant. Therefore, Ras-induced EMT-associated invasion in primed premalignant cells *via* post-transcriptional mechanisms (46). SCC-4 cells synthesize and release IL-6 independently, a process aided by TLR2/TLR6 agonists. In contrast to precancerous human tongue DOK cells, cancerous tongue SCC-4 cells exhibit a classic EMT profile (47). Beyond their immune function, CD4+ T cells are abundant in the dense stroma surrounding ductal epithelium in CP tissues associated with EMT. CD4+ T cells can induce EMT in premalignant cells (48). EMT is also facilitated by abnormal immune mediator expression in precancerous lesions. T cell dysfunction (49) or genetic changes (50) also contributes to developing immunosuppressive microenvironments during the malignant transformation of the oral mucosa by inducing EMT. The TGF-β (51) and PI3K-AKT (19) signaling pathways are critical in this transformation.

## Prospect

EMT is a common physiological process during embryogenesis, wound healing, fibrosis, tumorigenesis, and cancer metastasis. It has been artificially divided into three types based on different biological backgrounds, but the boundaries are not always clear. It is recommended to command the differential expressions of EMT in various situations to regulate the microenvironment to maintain equilibrium. EMT has applications in tissue regeneration and fibrosis inhibition, and we propose prospects. Concerning tooth tissue regeneration, two types of cells are indispensable: epithelial stem cells and mesenchymal stem cells (MSCs). They interact with and transform into one another under certain conditions. In dental tissue engineering, epithelial stem cells are primarily derived from embryonic tooth epithelium, while MSCs are derived from tooth germ and bone marrow stem cells.

### EMT and traditional approaches to tissue engineering

The interaction between seed cells, scaffold materials, and the microenvironment is central to current tissue engineering. Wang et al. invented the new concept of bio-root and saw it through to completion (52). Fruitful research on tooth regeneration has been published in the last few decades (53), and it is attractive to realize the clinical transformation of bio-root. However, common cell culture approaches are somewhat complicated for incorporating both epithelial stem cells and MSCs into the regenerative system, and increasing the workload. As a result, an EMT-induced culture system might be advantageous. Only one type of seed cell is added, and another type can be transferred from the original one *via* the regulation of cytokines and other signaling pathways. This method simplifies the operation and may provide a solution to the cell source shortage.

**Figure 3:**
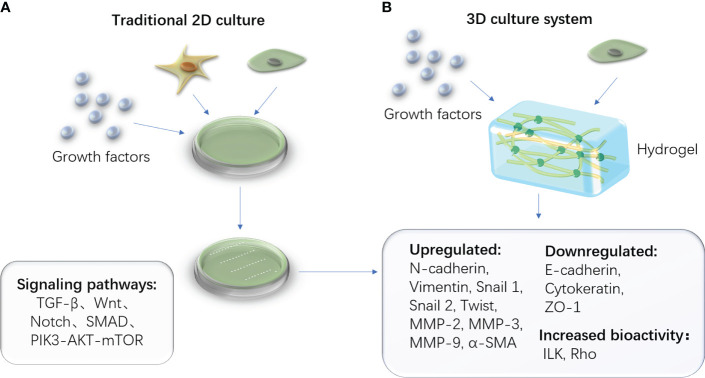
Traditional 2D cell culture system and advanced 3D cell culture system. **(A)** The schematic diagram of the traditional 2D culturing method, which contains stem cells and necessary cytokines in a culture medium. **(B)** The schematic diagram of a novel 3D culturing method in which the culture medium is replaced by the biomaterial hydrogel.

Recent studies on EMT have demonstrated that three-dimensional models can better simulate the extracellular matrix microenvironment, improve cell vitality, and reduce mortality than two-dimensional models, particularly for cartilage formation (54). The hydrogel is a porous, jelly-like structure that provides a biocompatible and non-toxic environment for cell growth, differentiation, and proliferation. It creates an environment for EMT/MET to occur by containing specific biomarkers and nutrient materials. The biocompatibility and histocompatibility of the ECM-derived hydrogel are higher (55). TGF-β1 induces EMT within the three-dimensional system for the decreased epithelial markers (E-cadherin), increased mesenchymal markers (Vimentin and α-SMA), and enhanced migratory and invasion capacity (56). (**Figure 3**) Furthermore, the treated dentin matrix, hyaluronic acid, PCL, and ceramics are widely used; they can be chosen during various tissue engineering study processes involving EMT (57–61).

However, the use of EMT in traditional tissue engineering is restricted by a lack of experimental conditions, unknown mechanisms, and operating techniques. Moreover, the condition of unclear regulation can contribute to tumor genesis and make the process uncontrollable. There remains much work to be done before we can put these considerations into practice.

### EMT in developmental biological regeneration

Unlike traditional tissue engineering, biological regeneration eliminates exogenous scaffolds and stimulates the organism’s regrowth. It is safer, easier, and has fewer side effects (62). On the other hand, the high demands for immunological microenvironments pose challenges. Bacterial pathogens, acid microenvironment, growth factors, proteins of primary signaling pathways, loss of apical-basal polarity, hypoxia, and other factors have all been implicated in the activation of EMT (18, 63–65). For instance, during long-term infection with the opportunistic pathogen *P. gingivalis*, human primary epithelial cells develop an EMT phenotype (66). Anaerobic periodontal pathogens have been shown to induce EMT in primary oral keratinocytes, destroying the periodontal barrier and contributing to periodontitis (63). EMT-associated transcription factors such as Slug, Snail, and Zeb1 showed significant increases in response to pathogen exposure. The treatment of EMT-related oral diseases may benefit from focusing on critical factors. The key to successful regeneration is determining how to precisely control these variables.

Coffee and EMT research has also received widespread attention. Coffee components can reverse EMT transitions or even rescue the functions of EMT inducers. For instance, Trigonelline extracted from natural coffee beans reduces renal fibrosis by inhibiting EMT (67). Chlorogenic acid derived from coffee has antitumor and anti-metastatic properties by interfering with the NF-κB/EMT signaling pathway (68). They exert pharmacological functions as EMT inhibitors, but more studies for clinical transformation are needed. Furthermore, because low PH promotes EMT, an alkaline diet and anti-acid drugs may effectively prevent EMT in the craniofacial tissues and organs (64).

Different signaling pathways and molecules regulate EMT in various biological or pathological processes. As a result, additional research into the individual mechanisms of each process is required to achieve precise control. Otherwise, it is considering how to find a balance between promoting practical functions and maintaining cell vitality. We are now at tipping in combining EMT and tissue engineering. They mutually aid in the advancement of regenerative medicine. The multi-discipline study is now the mainstream of research and has a promising future (69).

### Methods of preventing dysfibrosis

It is challenging to reduce the negative effects of dysfibrosis and keloids. As previously elucidated, oral mucosa has less keloid and fibrosis and heals wounds faster than the skin. The mechanisms of improved mucosal quality are primarily concluded as follows (9, 10): 1. Fewer pro-inflammatory factors and less inflammatory response; 2. Reduced recruitment of neutrophils, macrophages, and T cells after injury; 3. Certain microorganisms activate the immune system for wound healing cascade; 4. the suitable environment of saliva, which provides a biomimetic idea of hydrogel, applies to skin healing to accelerate the process (70). By inhibiting EMT, we may be able to design a type of epithelium with high regenerative capacity and self-repair without many keloids in the future.

The anti-EMT mainstream of reducing fibrosis during tissue regeneration or OSF (71). One solution is to improve EMT inhibitors such as phosphatase and tensin homolog, which inhibits the PI3K/AKT pathway to reduce hypertrophic scar fibroblast proliferation and eliminate keloid and fibrotic scars (72).

Another option is to use MSCs, which have anti-fibrotic, anti-oxidative, and angiogenesis properties, indicating that the cell is an ideal anti-fibrotic target.26 The functioning mechanism is attributed to inhibiting the TGF-β1 pathway *via* N-cadherin and vimentin downregulation (73, 74). MSCs are produced from epithelial cells through the continuous process of EMT. They have significantly higher levels of expression of several biomarkers, including CD105, CD73, and CD90 (75). Specific induction causes MSCs to differentiate into osteoblasts, adipocytes, and chondrocytes, differentiating into dental pulp stem cells (DPSCs), dental follicle stem cells (DFSCs), periodontal ligament stem cells (PDLSCs), and others. As a result, EMT serves as a unique source of seed cells for tissue regeneration.

MSCs are also critical in halting the process of OSF for immunomodulatory, anti-fibrotic, anti-oxidative, and angiogenic functions. Areca chewing can increase pro-inflammatory cytokines such as TNF-α and IL-6 in response to the microtrauma it causes, thereby promoting fibrosis progression (29). MSCs also suppress TNF-α expression *via* IL-10 secretion and downregulate TNF- α and IL-6 by inhibiting IFN-γ expression (76). MSCs also suppress the TGF-β pathway by secreting hepatocyte growth factor and TNF-stimulated gene 6 protein, which restores the TGF-β1/TGF-β3 balance for anti-fibrotic microenvironment production (77).

In this review, we present our expectations that in the future, we can apply EMT to regenerative medicine with or without scaffolding materials for profound progress in the following research. We owe the huge leap in the basic study of EMT to the progress made in the past few years. EMT significantly impact on scientific research and clinical transformation once the functional mechanisms are identified. We anticipate developing novel medicines for the treatment of EMT-related diseases in stomatology such as developmental malformation, wound repair, keloid and fibrosis, and other oral mucosa pathological alterations in the future.

## Author contributions

ZM, and TY wrote this manuscript. DL revised this manuscript. All authors contributed to the article and approved the submitted version.

## Funding

This research was funded by Tianjin Municipal Science and Technology Foundation grant number 21JCQNJC01190 and Tianjin Health Science and Technology Project grant number KJ20081. This research was also supported by the National Natural Science Foundation of China (82071079 to Dayong Liu)

## Acknowledgments

Authors thank the *Bullet Edits* for revising the English language. Authors also thank *Figdraw* of *Home for Researchers*, which supported Figures drawing.

## Conflict of interest

The authors declare that the research was conducted in the absence of any commercial or financial relationships that could be construed as a potential conflict of interest.

## Publisher’s note

All claims expressed in this article are solely those of the authors and do not necessarily represent those of their affiliated organizations, or those of the publisher, the editors and the reviewers. Any product that may be evaluated in this article, or claim that may be made by its manufacturer, is not guaranteed or endorsed by the publisher.
